# A Low-Profile Wideband Linear-to-Circular Polarization Conversion Slot Antenna Using Metasurface

**DOI:** 10.3390/ma13051164

**Published:** 2020-03-05

**Authors:** Jian Dong, Chang Ding, Jinjun Mo

**Affiliations:** 1 School of Computer Science and Engineering, Central South University, Changsha 410083, China; dongjian@csu.edu.cn (J.D.); dingchang@csu.edu.cn (C.D.); 2 School of Information and Communication, Guilin University of Electronic Technology, Guilin 541004, China

**Keywords:** metasurface, low-profile, wideband, polarization conversion, microstrip slot antenna

## Abstract

A new low-profile wideband linear-to-circular polarization conversion microstrip slot antenna based on a metasurface for C-band satellite communication applications is proposed in this paper. The metasurface basically consists of four unit cells with parasitic square cross gaps arranged in a 2 × 2 layout. By loading the metasurface on the microstrip slot antenna, linearly polarized (LP) waves from the source antenna are converted into circularly polarized (CP) waves. Then, by etching three more parasitic square cross gaps in the middle of the metasurface, enhanced impedance bandwidth and axial ratio bandwidth (ARBW) are achieved. Furthermore, an equivalent circuit and a phase analysis are presented to explain how a wide ARBW is realized by the metasurface. A final model with an overall size of 36 × 36 × 3.5 mm^3^ (approximately 0.65λ_0_ × 0.65λ_0_ × 0.06λ_0_ at 5.5 GHz) was designed and fabricated. The measured S_11_ bandwidth and 3 dB ARBW were 39.25% from 4.28 GHz to 6.37 GHz and 17.77% from 5.18 GHz to 6.19 GHz, respectively. As a result, the proposed antenna shows great potential for satellite communication applications due to its low profile and compact structure, wide impedance bandwidth, and wide axial ratio bandwidth.

## 1. Introduction

With the rapid development of wireless communication technology, there is a demand for the utilization of efficient antennas [1]. Recently, circularly polarized (CP) antennas have become an attractive choice for many communication systems, such as satellite communications and global positioning systems. This is mainly because CP antennas have numerous benefits over linearly polarized (LP) antennas, such as reducing the physical losses, providing anti-jamming, receiving an arbitrary polarization wave, and so on [2,3]. Microstrip patch antennas have been widely used in CP antennas due to their advantages in regard to their low cost, small size, simple structure, and easy integration [4]. Usually, a single-layer microstrip antenna can be circularly polarized by single-point feeding and multi-point feeding. The single-point feeding CP antenna has a simple structure but a narrow 3 dB axial ratio bandwidth (ARBW). The multi-point feeding CP antenna has the advantages of low cross-polarization, wide impedance bandwidth, and wide ARBW. However, it requires complicated feeding networks and increases the profile height of the antenna. Therefore, widening the circular polarization bandwidth while maintaining the antenna’s low profile is still a hot topic in current research [5].

As an artificial structural material, metasurfaces have received extensive research attention due to their novel electromagnetic properties that have not been found in nature [6]. New methods to design low-profile wideband CP microstrip antennas using metasurfaces have been demonstrated in the past few years, with resulting benefits over conventional CP microstrip antennas, such as reconfigurable polarization, miniaturization, wide bandwidth, and high gain [7]. Left- and right-hand circular polarization could be achieved by rotating the metasurface relative to the microstrip-fed slot antenna [8]. A metasurface with a unit cell consisting of five horizontal strips and a single diagonal strip realized the conversion from LP waves to CP waves [9], while a metasurface with square rings and splits achieved circular polarization and high gain [10]. By putting a double-stacked metasurface layer over a CP microstrip antenna, the ARBW was increased by 3% [11]. However, the sizes of the above designs were large, and their S_11_ bandwidth and ARBW were narrow, which limited the use of these antennas. Therefore, some metasurface CP microstrip antennas were designed to solve this issue of large size. In [5], a low-profile CP antenna was proposed by using a metasurface composed of square units with truncated corners. In [7], a miniaturized antenna loading capacitive strips on the corner-truncated patch was investigated. In [12], by introducing a triangular interdigitated capacitor and diagonal slot loading to the metasurface, an advanced metasurface CP antenna with a compact footprint and improved 3 dB ARBW was constructed. In [13], by directly placing a circularly shaped metasurface atop a slot antenna, a compact, low-profile, polarization-reconfigurable antenna was presented. In [14], the metasurface of a 3 × 4 rectangular patch array was utilized to design a low-profile broadband circularly polarized antenna for C-band satellite communication. These designs reduced the overall size slightly at the cost of reduced S_11_ bandwidth and ARBW. In addition, some other metasurface CP microstrip antennas were introduced to broaden the ARBW. A corner-truncated square metasurface [15] and a 7 × 7 smaller patch array metasurface [16] were used to construct wideband CP slot antennas. These designs realize a wide S_11_ bandwidth and ARBW but still have a large-sized structure.

This paper designs a new low-profile wideband linear-to-circular polarization conversion antenna for satellite communications by placing a “parasitic square cross gap” metasurface on a slot microstrip antenna. In Section 2, the geometry of the proposed antenna is presented, followed by polarization reconfigurability. Next, an equivalent circuit and a phase analysis are presented to explain how the proposed antenna converts LP waves to CP waves and obtains a wide ARBW. In Section 3, the parameters affecting the ARBW of the antenna are optimized. In Section 4, the proposed CP metasurface antenna is fabricated and measured. The measured results are in good agreement with the simulated results. Finally, in Section 5, the conclusions are presented.

## 2. Design and Analysis of the Proposed Antenna

### 2.1. Design of the Metasurface Antenna

**Figure 1:**
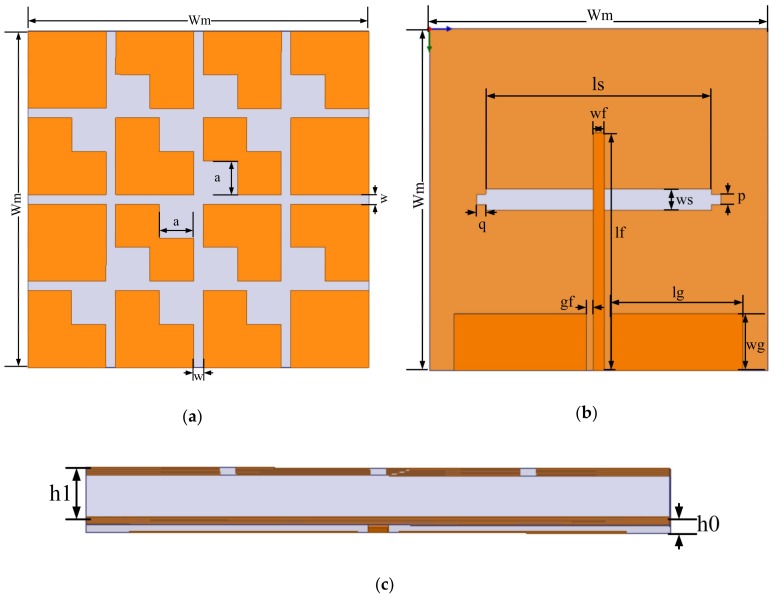
Geometry of the proposed antenna: (**a**) top view; (**b**) rear view; (**c**) side view.

The final low-profile wideband CP metasurface antenna is presented in Figure 1. It is formed of two parts and three layers. Both of the parts were designed on an inexpensive FR4 substrate with a dielectric constant of ε_r_ = 4.4 and loss tangent of δ = 0.02. The bottom part is the slot microstrip LP antenna, i.e., the source antenna, and the top part is the loaded metasurface. The thicknesses of the bottom substrate h_0_ and top substrate h_1_ are 0.5 mm and 3 mm, respectively. They were designed to have the same area of Wm × Wm = 36 × 36 mm^2^. The top layer is the metasurface, which consists of four unit cells arranged in a 2 × 2 layout on a substrate with seven parasitic square cross gaps. The middle layer is the metallic ground plane with an air slot of ls × ws = 28 × 2.2 mm^2^ and two slots of p × q = 1.1 × 1 mm^2^. A step slot was used for better impedance matching [17]. To integrate the metasurface with the slot coupling antenna and excite the slot antenna radiating properly, the bottom layer adopts a coplanar waveguide (CPW) feeding line. All parameter values of the proposed CP metasurface antenna are tabulated in Table 1.

**Figure 2:**
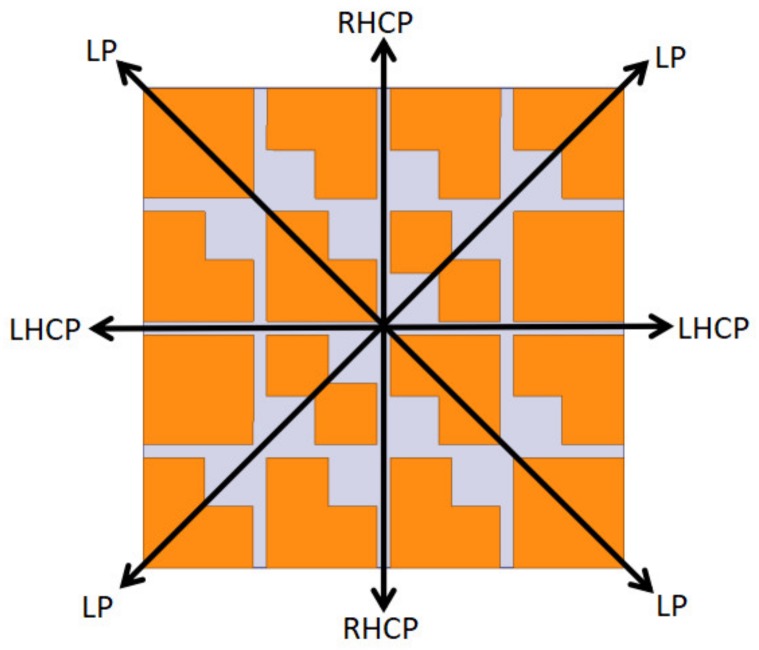
Antenna polarization at different rotation angles.

Polarization reconfigurability of the proposed metasurface antenna can be accomplished by rotating the metasurface relative to the source antenna. The rotation angle was studied with respect to the y-axis, as shown in Figure 2. It can be seen that, if the rotation angle is 0° or 180°, the metasurface antenna gives a right-hand circular polarization (RHCP) wave, whereas, for 90° or 270°, it gives left-hand circular polarization (LHCP). If the rotation angle is 45° or 135°, the antenna is linearly polarized [13]. RHCP without a rotation angle is illustrated as an example in the following analysis for convenience of explanation.

### 2.2. Mechanism of Circular Polarization

**Figure 3:**
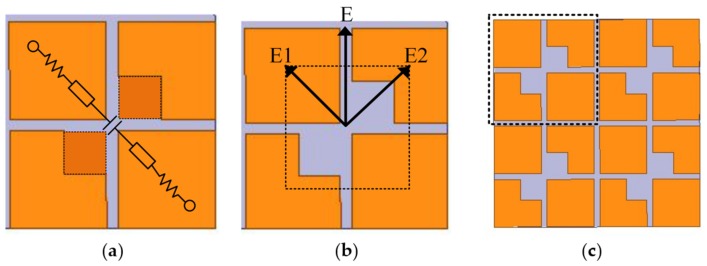
The mechanism of generating circularly polarized waves from linearly polarized waves for the corner-cut square metasurface (**a**) an equivalent circuit of the metasurface unit cell; (**b**) an electric field distribution of the metasurface unit cell; (**c**) the basic metasurface.

An equivalent circuit of the metasurface unit cell is shown in Figure 3a. When there are no parasitic squares at the diagonal corners of the cross, the orthogonal components will have the same impedance because of the symmetrical structure. **E**_1_ and **E**_2_ treat the metasurface as an equivalent RLC circuit, as shown in Figure 3b, the impedance of which can be written as Equation (1):

(1) $$Z=2R+j\omega (2L)+\frac{1}{j\omega C},$$

where *R* and *L* denote the resistance and inductance, respectively. C represents the capacitance created by the cross gaps. **E**_1_ and **E**_2_ can be seen as two different impedances Z_1_ and Z_2_. When the corners are truncated, the cross gaps increase, resulting in Z_1_ being more capacitive than Z_2_. Therefore, the phase difference between Z_1_ and Z_2_ can be varied by changing the size of the parasitic squares. When |Z_1_| = |Z_2_| and ∠Z_1_−∠Z_2_ = 90°, then |**E**_1_| = |**E**_2_| and ∠**E**_1_−∠**E**_2_ = 90°, and circular polarization can be realized [8]. Therefore, the corner-cut square metasurface unit cell of Figure 3b was arranged in a 2 × 2 layout to form the basic metasurface of Figure 3c. 

According to the basic theory of electromagnetic fields, circularly polarized waves can be decomposed into two linearly polarized waves that are orthogonal to each other, having the same amplitude and a phase difference of 90°. Therefore, these two conditions must be met to achieve linear-to-circular polarization conversion. As shown in Figure 3b, the linearly polarized wave is incident along the **E** direction, and the angle between this and **E**_1_ is θ. When θ = 45° or θ = 135°, the linear polarization wave can be decomposed into two orthogonal linear polarization components with equal amplitude. If θ = 45° is used as an example for analysis, the electric field of the incident wave can be expressed as Equation (2) [18,19]:

(2) $$\vec{E}=\left|{\vec{E}}_{1}\right|e^{j\theta_{1}}+\left|{\vec{E}}_{2}\right|e^{j\theta_{2}}=\frac{\sqrt{2}}{2}\left({\vec{E}}_{1},{\vec{E}}_{2}\right)\left(\begin{array}{c} 1\\ 1 \end{array}\right).$$

Let φ_1_ and φ_2_ be the reflection phases of the unit in directions **E**_1_ and **E**_2_. If |φ_1_−φ_2_| = 90°, then Equation (2) can be rewritten as Equation (3):

(3) $${\vec{E}}_{c}=\frac{\sqrt{2}}{2}\left({\vec{E}}_{1},{\vec{E}}_{2}\right)\left(\begin{array}{c} 1\\ \pm j \end{array}\right).$$

Furthermore, the electric field can be rewritten in terms of the metasurface of the two circularly polarized components **E**_1_ and **E**_2__;_ thus, the axial ratio (AR) can be easily determined according to Equation (4) [18]:

(4) $$AR_{\mathrm{dB}}=20\log\left(\frac{\left|{\vec{E}}_{1}\right|+\left|{\vec{E}}_{2}\right|}{\left|{\vec{E}}_{1}\right|-\left|{\vec{E}}_{2}\right|}\right).$$

**Figure 4:**
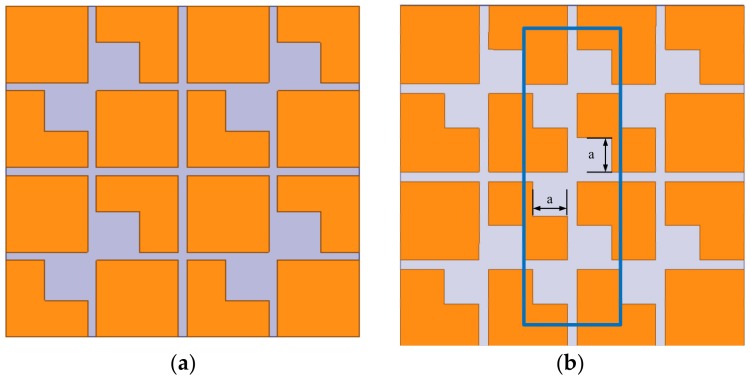
Geometry of the metasurface with (**a**) four parasitic square cross gaps; (**b**) seven parasitic square cross gaps.

**Figure 5:**
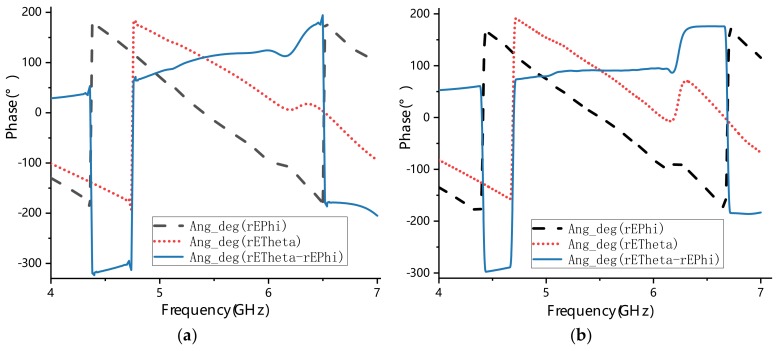
Phases of **E**_1_, **E**_2_, and their differences in (**a**) the metasurface with four parasitic square cross gaps and (**b**) the metasurface with seven parasitic square cross gaps.

The above analysis explains how circularly polarized waves are generated. From the basic metasurface with four parasitic square cross gaps shown in Figure 4a, we inferred that circularly polarized performance of the antenna could be enhanced by adding more metasurface unit cells of Figure 3b. To keep the miniaturization of the antenna, we proposed a new metasurface with seven parasitic square cross gaps shown in Figure 4b. Then, the phase analysis was used to verify the circularly polarized performance of two metasurfaces shown in Figure 4. By simulating these two metasurfaces, we can obtain phases of the **E**_1_ and **E**_2_ waves. The phase difference between **E**_1_ and **E**_2_ is shown in Figure 5. It can be seen that the phase difference of the metasurface with seven parasitic square cross gaps is much closer to 90° than that of the metasurface with four parasitic square cross gaps. Therefore, we chose the metasurface with seven parasitic square cross gaps as the final design.

## 3. Parametric Analysis

**Figure 6:**
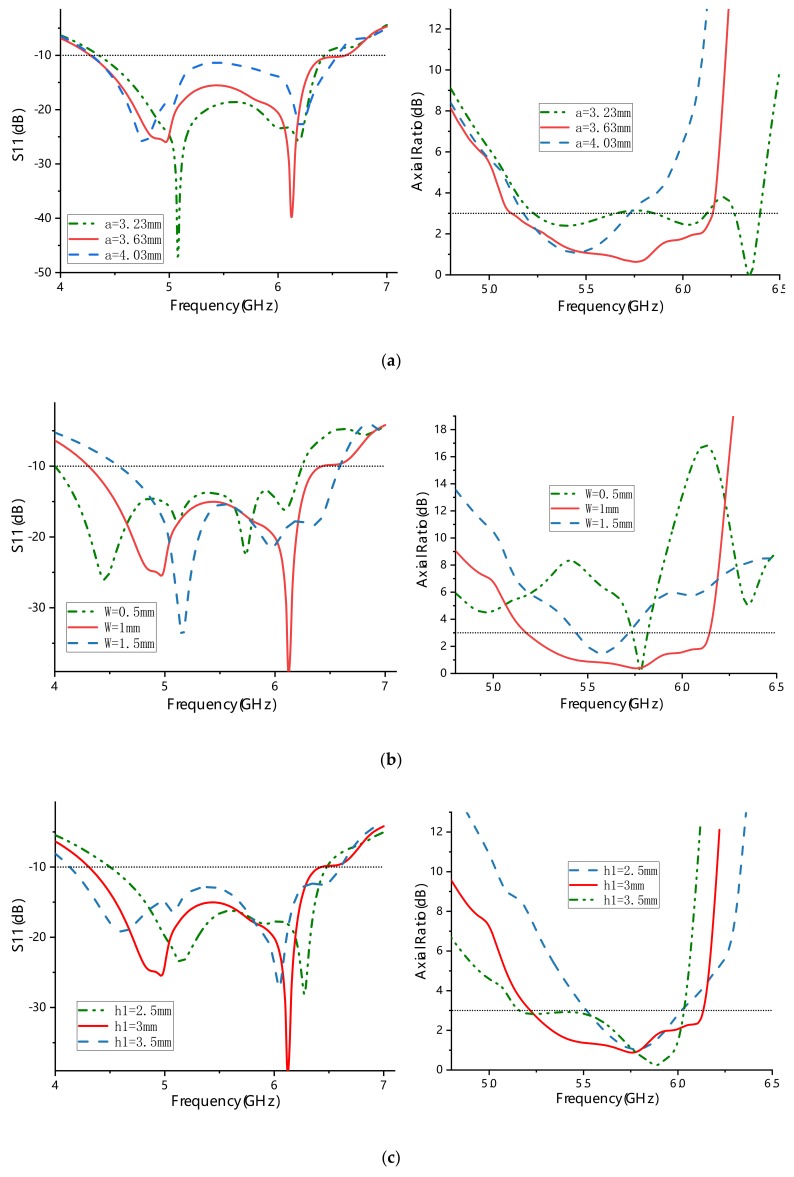

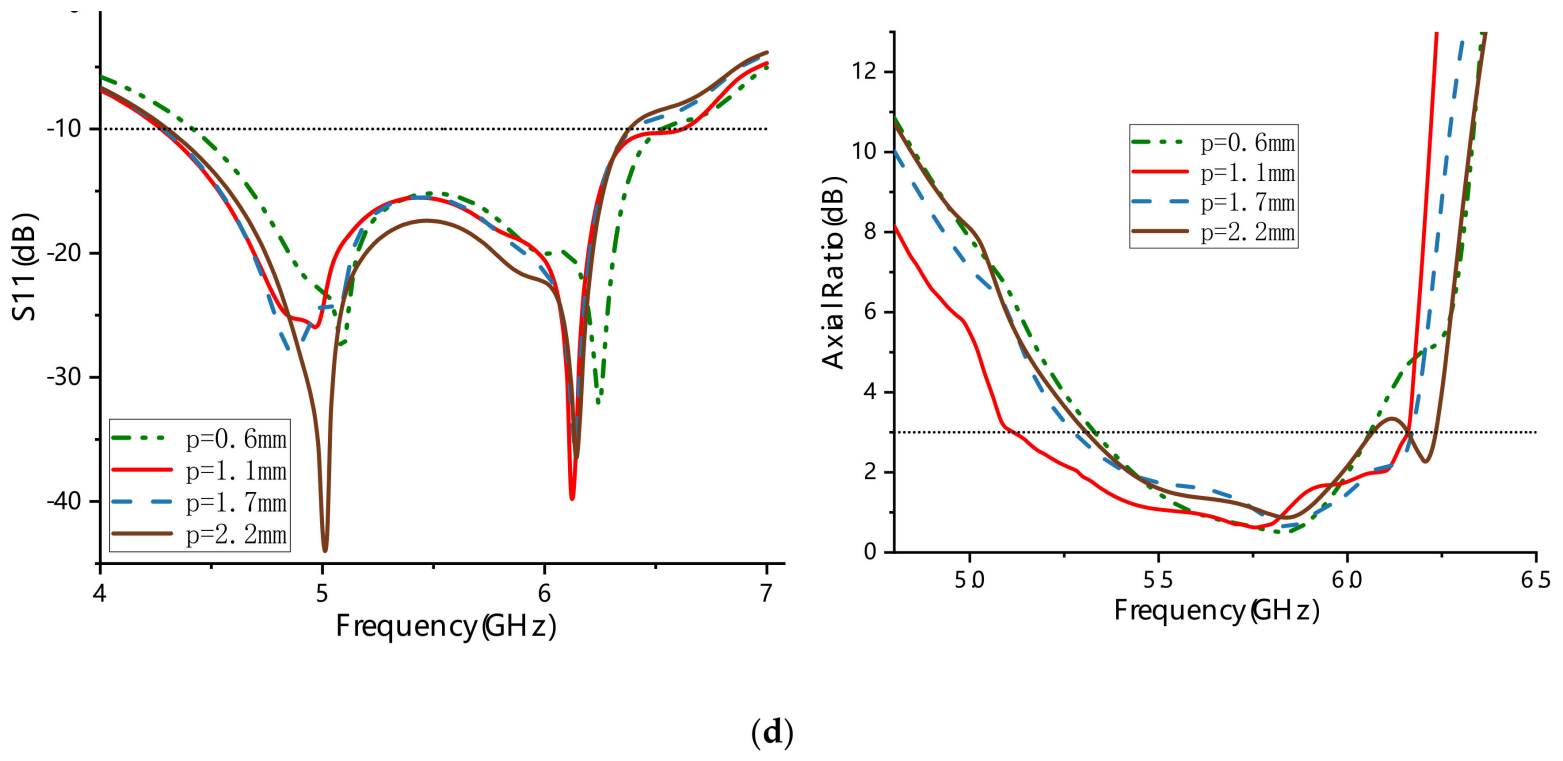
Axial ratio (AR) and S_11_ characteristics of the proposed metasurface antenna with different parameters: (**a**) a; (**b**) w; (**c**) h_1_; (**d**) p.

The dimensional parameters of the metasurface and the source antenna should be optimized to realize wideband circular polarization characteristics of the proposed antenna. Through simulation analysis, it was found that the two main parameters of the metasurface (a,w) and the thickness of the top substrate (h_1_) have an obvious influence on the axial ratio performance of the antenna. As illustrated in Figure 6a, when a is decreased from 3.63 mm to 3.23 mm, the frequency dip shifts up to 6.4 GHz with AR = 0 dB. However, from 5.25 GHz to 6.3 GHz, the AR value fluctuates around 3 dB, which leads to a narrow ARBW. As a is increased to 4.03 mm, the frequency dip drops to 5.45 GHz, and its ARBW is about half that at a = 3.63 mm. Figure 6b shows that when the value of the unit cell spacing w is larger than or less than 1 mm, the axial ratio values increase, and the ARBWs narrow. The ARBWs can be adjusted by changing the values of a and w, mainly because the equivalent impedance of the metasurface equivalent circuit is thus changed. When the value of the thickness of the top substrate h_1_ is larger than or less than 3 mm, the ARBWs are narrowed, as illustrated in Figure 6c. When the value of p is larger than or less than 1.1 mm, the ARBW is narrower than that at p = 1.1 mm, as shown in Figure 6d. In addition, when p = 2.2 mm, the shape of the middle metal plate groove is a rectangle. A hump band (with an axial ratio value of >3 dB) appears around 6.1 GHz, which makes the ARBW narrow. When the parameter values of a, w, h_1_, and p are adjusted to optimize ARBW, the corresponding S_11_ bandwidth experiences only little change. Therefore, the optimum ARBW combination of a = 3.63 mm, w = 1 mm, h_1_ = 3 mm, and p = 1.1 mm was chosen for a wide ARBW and good impedance matching. 

## 4. Measured Results Analysis

**Figure 7:**
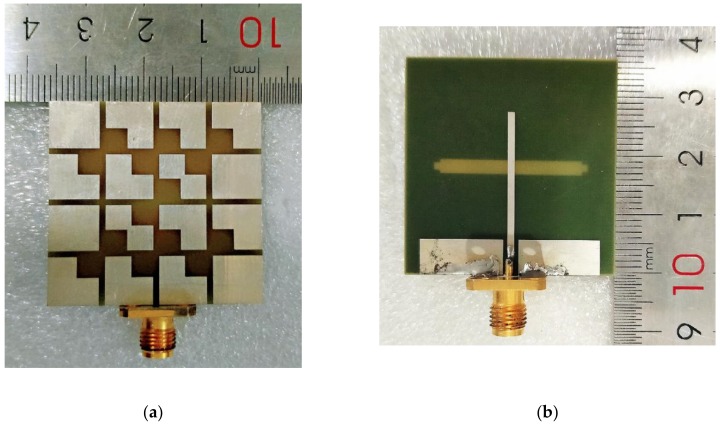
Photographs of the fabricated metasurface antenna: (**a**) top view; (**b**) rear view.

**Figure 8:**
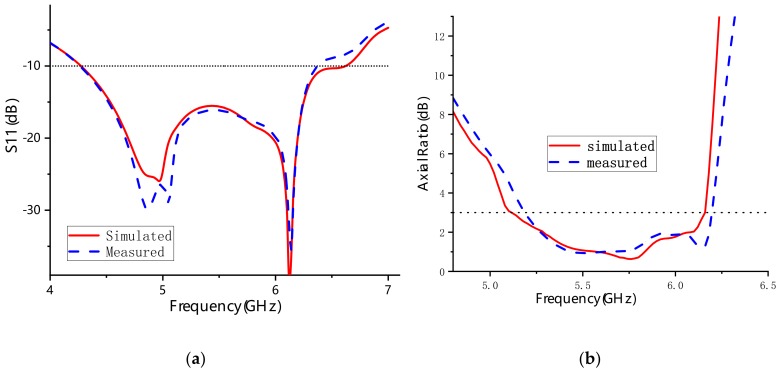
Simulated and measured characteristics of the proposed antenna: (**a**) S_11_; (**b**) AR.

To further verify the impedance, axial ratio, and radiation performance of the proposed design, the metasurface antenna was fabricated using a common printed circuit board (PCB) fabrication approach, as shown in Figure 7. Figure 8 illustrates the impedance bandwidth and 3 dB axial ratio bandwidth obtained by simulation and measurement in the broadband range. The simulated results showed that the S_11_ bandwidth and 3 dB ARBW of the proposed metasurface antenna were 42.94% from 4.28 GHz to 6.62 GHz and 18.83% from 5.1 GHz to 6.16 GHz, respectively. The fabricated antenna had a −10 dB impedance bandwidth of 39.25% (4.28–6.37 GHz) and a 3 dB axial ratio bandwidth of 17.77% (5.18–6.19 GHz). The small variation between the measured and simulated results probably comes from fabrication tolerances and limitations of the measured environment. 

**Figure 9:**
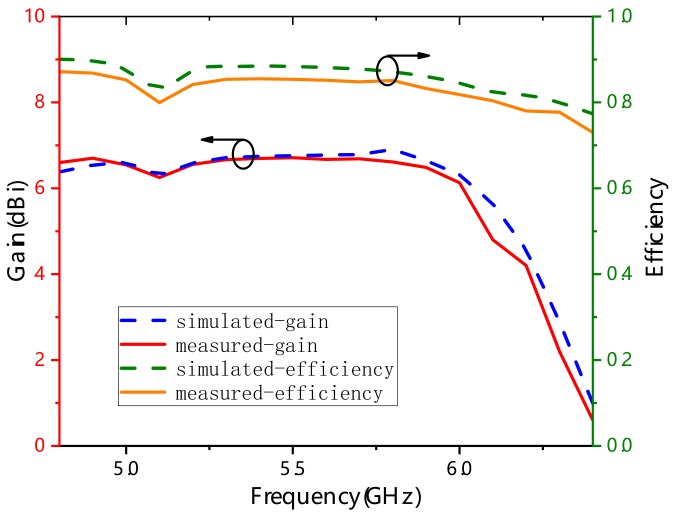
Simulated and measured gain and efficiency of the proposed antenna.

Figure 9 illustrates the gain and efficiency characteristics of the proposed antenna obtained by simulation and measurement. Over the bandwidth ranging from 5 GHz to 6 GHz, the measured gain and radiation efficiency fluctuated slightly around 6.8 dB and 88%, respectively. The slight differences between measured and simulated results may come from the increased actual dielectric constant of the substrate, the fabrication, or assemble tolerance of the antenna.

**Figure 10:**
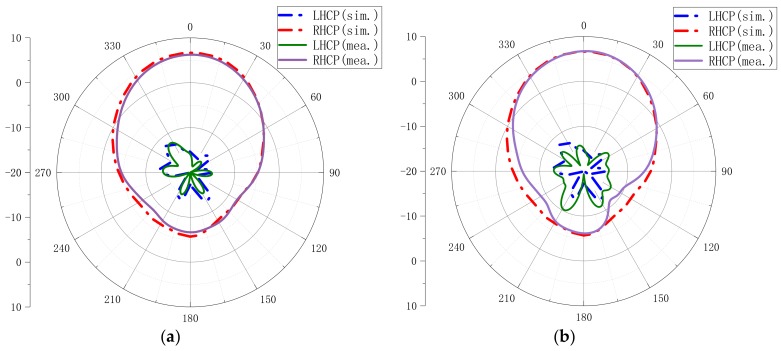
Comparison of simulated and measured radiation patterns at 5.5 GHz: (**a**) xoz-plane; (**b**) yoz-plane.

Figure 10 shows the simulated and measured radiation patterns of the xoz-plane and yoz-plane at 5.5 GHz. It can be observed that the measured and simulated results basically agree with each other. The proposed antenna has a low LHCP in both the xoz-plane and the yoz-plane. The measured LHCP level was <−15 dB in the xoz-plane and <−10 dB in the yoz-plane. The measured RHCP level was approximately from −8 dB to 6 dB in the xoz-plane and approximately from −11 dB to 7 dB in the yoz-plane. The RHCP level was higher than the LHCP level, which means that the proposed antenna is a right-hand circular polarized antenna. 

Table 2 summarizes the key performance indicators of recently reported CP antennas based on metasurfaces for comparison. It can be seen that both the S_11_ bandwidth and ARBW of the proposed CP metasurface antenna are much wider than those of the designs in [8,9,10,11,20]. Moreover, the size of the proposed antenna is much smaller in comparison. Furthermore, the size of the proposed antenna is a little larger than those of the designs in [5,7,12,13,14], but with a wider S_11_ bandwidth and ARBW. Although the ARBWs in [15,16] are a little wider than that of the proposed antenna, their S_11_ bandwidths and overall sizes are worse than those of the proposed antenna. 

## 5. Conclusions

A new low-profile wideband circularly polarized microstrip slot antenna based on a metasurface was presented herein. The metasurface was used to realize reconfigurable polarization and widen the working frequency bandwidth. The proposed CP metasurface antenna has a compact structure with a low profile of 0.06λ_0_. Simulated results showed that the S_11_ bandwidth and 3 dB ARBW of the proposed metasurface antenna were 42.94% from 4.28 GHz to 6.62 GHz and 18.83% from 5.1 GHz to 6.16 GHz, respectively. The measured S_11_ bandwidth and 3 dB ARBW were 39.25% from 4.28 GHz to 6.37 GHz and 17.77% from 5.18 GHz to 6.19 GHz, respectively. The proposed CP metasurface antenna exhibits good prospects for C-band satellite communication applications with its compact structure, wide impedance bandwidth, and axial ratio bandwidth.

## Figures and Tables

**Table 1:** Design parameters of the proposed circularly polarized metasurface antenna (in mm).

Wm	w	a	ls	ws	lf	wf	lg	wg	p	q	gf	h_1_	h_0_
36	1	3.63	24	2.2	7	1.2	14	6	1.1	1	0.7	3	0.5

**Table 2:** Performance comparison of recently reported CP antennas based on metasurfaces.

REF	Size (λ_0_^3^)	*f*_o_ (GHz)	−10 dB S_11_ BW (%)	3 dB ARBW (%)	Remarks
Ref. [8]	0.96 × 0.96 × 0.06	2.4	22.2	8.2	large size, narrow S_11_ bandwidth, and narrow ARBW
Ref. [9]	0.8 × 0.98 × 0.02	4.2	—	12	large size and narrow ARBW
Ref. [10]	1.87 × 1.87 × 0.6	7.45	6.87	6.87	large size, narrow S_11_ bandwidth, and narrow ARBW
Ref. [11]	0.94 × 0.94 × 0.61	2.2	11.85	12.04	large size, narrow S_11_ bandwidth, and narrow ARBW
Ref. [20]	3.14 × 3.14 × 0.1	11.8	13.8	6.11	large size, narrow S_11_ bandwidth, and narrow ARBW
Ref. [5]	0.4 × 0.4 × 0.03	2.49	17	7.2	small size, narrow S_11_ bandwidth, and narrow ARBW
Ref. [7]	0.58 × 0.58 × 0.043	3.5	23.39	8.5	small size, narrow S_11_ bandwidth, and narrow ARBW
Ref. [12]	0.61 × 0.52 × 0.05	4	16	10	small size, narrow S_11_ bandwidth, and narrow ARBW
Ref. [13]	0.36 × 0.81 × 0.035	3.5	24	11.4	small size, narrow S_11_ bandwidth, and narrow ARBW
Ref. [14]	0.6 × 0.49 × 0.07	5.25	33.7	16.5	small size, narrow S_11_ bandwidth, and narrow ARBW
Ref. [15]	0.71 × 0.71 × 0.06	5.5	33.6	18.2	large size, narrow S_11_ bandwidth, and wide ARBW
Ref. [16]	1.07 × 0.82 × 0.066	4.1	34.2	19.5	large size, narrow S_11_ bandwidth, and wide ARBW
**Proposed**	0.65 × 0.65 × 0.06	5.5	39.25	17.77	small size, wide S_11_ bandwidth, and wide ARBW
